# The Antidepressant Effect of* Angelica sinensis* Extracts on Chronic Unpredictable Mild Stress-Induced Depression Is Mediated via the Upregulation of the BDNF Signaling Pathway in Rats

**DOI:** 10.1155/2016/7434692

**Published:** 2016-08-25

**Authors:** Jun Shen, Junjian Zhang, Min Deng, Yue Liu, Yuan Hu, Lei Zhang

**Affiliations:** Department of Neurology, Zhongnan Hospital, Wuhan University, No. 169, Donghu Road, Wuhan, Hubei 430071, China

## Abstract

*Angelica sinensis* (AS), a traditional Chinese herbal medicine, has pharmaceutical effects on menstrual illness, cerebrovascular diseases, cardiovascular diseases, and cognitive impairments. However, until recently, few studies had explored its antidepressant effect. The current study attempts to investigate the effect of AS extracts on chronic unpredictable mild stress- (CUMS-) induced depression in rats. Male SD rats were exposed to a CUMS-inducing procedure for 5 weeks, resulting in rodent depressive behaviors that included reduced sucrose consumption and lessened sucrose preference ratios in sucrose preference test, prolonged immobility times and decreased struggling time in force swim test, and decreased locomotor activity in open field test. Moreover, the expression of brain derived neurotrophic factor (BDNF) and the phosphorylation of cAMP-response element binding protein (CREB) and extracellular signal-regulated protein kinase (ERK 1/2) were markedly decreased in the hippocampus in depressed rats. However, chronically treating the depressed rats with AS (1 g/kg) normalized their depression-related behaviors and molecular profiles. In conclusion, in the present study, we show that AS extracts exerted antidepressant effects that were mediated by the BDNF signaling pathway: in AS-treated depressed rats, the expression of the BDNF protein and the phosphorylation of its downstream targets (ERK 1/2, CREB) were upregulated in the hippocampus.

## 1. Introduction

Depression is one of the most common psychiatric disorders, affecting approximately 20% of the worldwide population [1, 2]. According to the World Health Organization, by the year 2020, depression will be the second leading cause of illnesses that result in disability [3]. The corn-related symptoms of depression include anhedonia, loss of interest, low spirit, and feelings of worthlessness. A growing amount of evidence supports the notion that neurotransmitters deficiencies are an extraordinarily important etiology in depression; monoamines oxidase inhibitors and selective serotonin reuptake inhibitors (SSRIs) are therefore widely prescribed for patients with depression. However, it always takes a few weeks for these drugs to induce an antidepressant effect in patients, and meanwhile, the side effects of the drugs, including dry mouth, constipation, bladder problems, sexual problems, dizziness, sleepiness, and nausea, are frustrating [4]. Most importantly, approximately one-third of patients do not experience a relief of symptoms even when they are treated with antidepressants.

In addition to the neurotransmitters hypothesis, neurotrophic hypothesis of depression recently gained a large amount of attention [5]. Among neurotrophic factors, BDNF has become a hot topic in human research [6–8]. Some preclinical studies have confirmed that BDNF expression in the hippocampus is downregulated when rodents are subjected to CUMS, while antidepressants, exercise, and acupuncture normalized BDNF protein expression in the hippocampus and thereby alleviated the depressive state in rodents [9–11]. When the BDNF gene was knocked out in rats or the rats were injected with a BDNF receptor antagonist, hippocampal neurogenesis was clearly suppressed, and depressive symptoms nearly always reoccurred [12, 13]. Clinical studies shed light on the fact that serum levels of BDNF are decreased in depressed patients, whereas chronically administering antidepressants ameliorated depressive symptoms and increased serum levels of BDNF [14, 15]. Moreover BDNF gene (Val66Met polymorphism) was shown to be a strong risk factor for developing major depression [16]. Postmortem studies of depressed patients revealed that their hippocampal volume was reduced, their neurons had become atrophied, and their synaptic plasticity was impaired [17]. All of these studies indicated that BDNF plays critical roles in the pathogenesis of depression.

The traditional Chinese medical herb AS was first described in “Shennong's Herbal Classic.” It was initially used to relieve menstrual symptoms and cerebrovascular and cardiovascular diseases [18–20]. More recently, a clinical study showed that AS and* Matricaria chamomilla* (Climex) alleviated women's menopausal symptoms, including anxiety, skin flushes, menstrual disorders, sleep disorders, and fatigue, symptoms that have been shown to be predictors of depression and anxiety [21]. Meanwhile, the herb Jia-Wei-Xiao-Yao-San was found to exert a protective effect against major depressive disorder and sleep disorders in a clinical study [22]. A preclinical study showed that the formula Dang-Gui-Shao-Yao-San exerted a beneficial effect against depression [23]. The major active component in Jia-Wei-Xiao-Yao-San, Dang-Gui-Shao-Yao-San, and Climex compounds is AS. Moreover, our previous study showed that AS relieved chronic restraint stress-induced alterations in cognitive associated behaviors by increasing synaptic plasticity, upregulating hippocampal BDNF expression, and restoring impaired LTP and memory functions in an animal model. However, the mechanism by which AS extracts exert their antidepressant effect has not been fully explored. In this study, we attempt to explore the mechanism underlining the effect of AS extracts on CUMS induced depression in rats by assaying hippocampal BDNF protein expression levels and the phosphorylation of CREB and ERK 1/2.

## 2. Materials and Methods

### 2.1. Animals

Male Sprague-Dawley rats weighing 140–160 g were purchased from Beijing Vital River Laboratory Animal Company. Before starting the experiments, all of the rats were acclimated to the novel experimental environment (12 h light dark cycle, 22 ± 2°C of room temperature) for one week with free access to food and water.

All animals were cared for according to the National Institutes of Health Guide and the experiment was approved by the Animal Ethics Committee of the Medical School of Wuhan University.

### 2.2. Preparation of AS Extracts

A standardized extract from dry roots of* Angelica sinensis* (Oliv.) Diels was provided by the Jiangyin Tianjiang Pharmaceutical Co., Ltd. (Jiangsu Province, China, batch number: 1001002694). The extracts were obtained from sliced AS roots (1000 g) by washing the samples in 75% ethanol three times in a reflux condenser for 4 h; temperature of the room was set at 26 ± 2°C. The solutions were combined, concentrated, and filtered under reduced pressure at 50°C. The ethanol extract was then lyophilized into powders (76.4 g; 7.64% yield).

**Figure 1:**
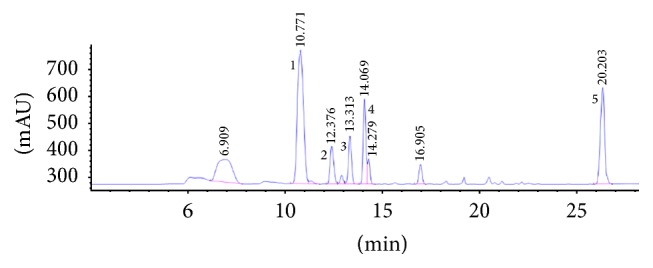
HPLC chromatograms of ferulic acid (1), butylphthalide (2), E-ligustilide (3), E-butylidenephthalide (4), and E-ligustilide (5).

HPLC confirmed that the extracts contained five active compounds, including ferulic acid, butylphthalide, Z-ligustilide, E-ligustilide, and E-butylidenephthalide. These results were consistent with previous studies [24, 25]. HPLC was performed using Agilent Technologies 1220 Infinity LC. The following parameters were used: EtOAc : MeOH = 75 : 25 and flow rate = 1.0 mL/min. The HPLC results of AS extracts are showed in Figure 1.

### 2.3. Drug Administration

The positive control drug fluoxetine was provided by Lilly Suzhou Pharmaceutical Co., Ltd. (Jiangsu Province, China). The rats were randomly divided into four groups: control, CUMS, Flu, and AS. All rats were acclimated for one week, and then all rats except the control group rats were subjected to the CUMS procedure for 2 consecutive weeks. Then in the next week, all rats were subjected to depression-related behavioral tests. During the 3 subsequent consecutive weeks, 1 g/kg AS extract and 10 mg/kg Flu were diluted in water and administered to the AS group and the Flu group, respectively, via gastric gavages every day at 1 h before CUMS procedure. The dose of 1 g/kg AS extract and 10 mg/kg Flu was used according to previous studies [26, 27]. The rats in the CUMS group were administered an equal amount of water using the same method during the last 3 weeks. The experimental design is shown in Table 1.

### 2.4. Chronic Unpredictable Mild Stress

CUMS was performed according to Ducottet et al., with minor modifications [28]. The procedure contains different mild stressors such as water deprivation for 24 h, food deprivation for 24 h, tail pinch for 1 min (clip used 1 cm near the end of the tail), physical restraint for 2 h (plastic bottles), cold water for 5 min (5°C), hot water for 5 min (45°C), overnight illumination, soiled cage for 24 h (200 mL water and 100 g sawdust mixed together), or cage tilted (45°) for 7 h. The stressors were randomly performed every day for 5 weeks except water or food deprivation for two consecutive days. The control group rats were housed in another room to separate them from the disturbances that the experimental group of rats was exposed to.

### 2.5. Sucrose Preference Test

The procedure was performed as previously described [29]. Two bottles of 1% sucrose solution (w/v) were placed on each cage. The rats had free access to drink from these bottles for the first 24 h. Then, during the next 24 h, one bottle was filled with water while the other continued to contain 1% sucrose solution. The positions of the two bottles were exchanged after 12 h to avoid the influence of bottle position. All of the rats were deprived of water for the last 23 h of the experiment. The sucrose preference test was conducted from 9 am to 10 am for every rat that was housed alone, during which they had free access to 100 mL of water and 100 mL of a 1% sucrose solution. The amount of water and sucrose solution that were consumed was recorded, and the sucrose preference rate was calculated as consumed sucrose solution/(consumed water + consumed sucrose solution).

### 2.6. Forced Swim Test

The forced swim test was performed as previously described [30]. On the first day, every rat was subjected to swim in a plastic cylinder (60 cm in height and 20 cm in diameter) that was filled with 30 cm of water (25°C) for 15 min. On the next day, each rat was put into the water again for 5 min and video-taped. The amount of time spent immobile was scored as depression when they floated with their heads above the water and showed no sign of struggling.

### 2.7. Open Field Test

Open field test was performed as previously described [31]. The open field apparatus was a black box (height: 40 cm, length: 100 cm, and width: 100 cm) containing 25 equal squares regions that were defined by lines on the floor of the box. Every rat was placed into the center of the box and provided with free access to any location in the floor for 5 min. The rat was then removed from the box, and the floor was cleaned with ethanol to eliminate odors. The number of crossings and rearing that were observed during the test were recorded for every rat.

### 2.8. Western Blot Analysis

On the day after the last behavior test, all of the rats were anesthetized using 10% chloral hydrate (350 mg/kg, intraperitoneal injection) and then decapitated. The whole brain was separated from the skull, and the hippocampus was immediately dissected while the brain was on ice surface. The hippocampus was then frozen in liquid nitrogen and stored at −80°C in a freezer until used. The hippocampus was homogenized in ice-cold RIPA buffer and then centrifuged at 12,000 rpm at 4°C for 15 min and supernatant was collected. The protein concentration in the supernatant was measured using a BCA protein assay kit. Electrophoresis was performed using SDS-PAGE on 15% polyacrylamide gels. The proteins were then transferred to polyvinylidene difluoride membrane and probed using the following primary antibodies: anti-BDNF (1 : 500; Millipore), anti-p-CREB (1 : 400; Millipore), and anti-p-ERK (1 : 1000). The membranes were then washed and incubated with HRP-conjugated anti-rabbit IgG. The imaging system was used to measure the optical densities of the immune-reactive bands.

### 2.9. Statistical Analysis

All results are presented as mean ± SD. SPSS 19.0 was used to analyze the data. One-way ANOVA followed by an LSD test was used for multiple post hoc comparisons of means. Statistical differences were considered as the value of *p* < 0.05.

## 3. Results

### 3.1. Effects of AS on Body Weight

**Figure 2:**
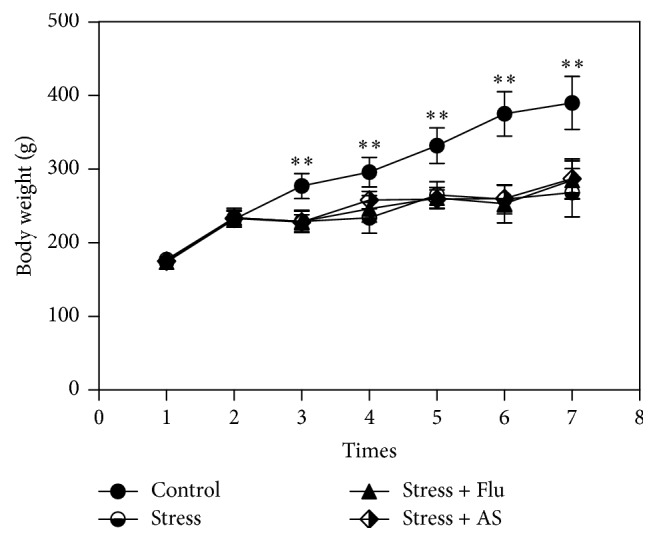
Effects of AS on body weight. The body weight of all rats which were recorded seven times at every weekend. The results were represented as mean ± SD (*n* = 8 for each group). ^*∗∗*^ *p* < 0.01 compared with stress group.

The results were shown in Figure 2. There was no statistical differences between the body weights of the rats in the four groups at the beginning of the experiment (*p* > 0.05). Beginning in the third week, the rats in the CUMS group showed less body weight gain than the rats in the control group (*p* < 0.01), and similar results were observed between the AS group and the control group (*p* < 0.01). However, there was no significant difference in body weight across the rats in the fluoxetine, AS, and CUMS groups (*p* > 0.05).

### 3.2. Effects of AS on Sucrose Preference Tests

**Figure 3:**
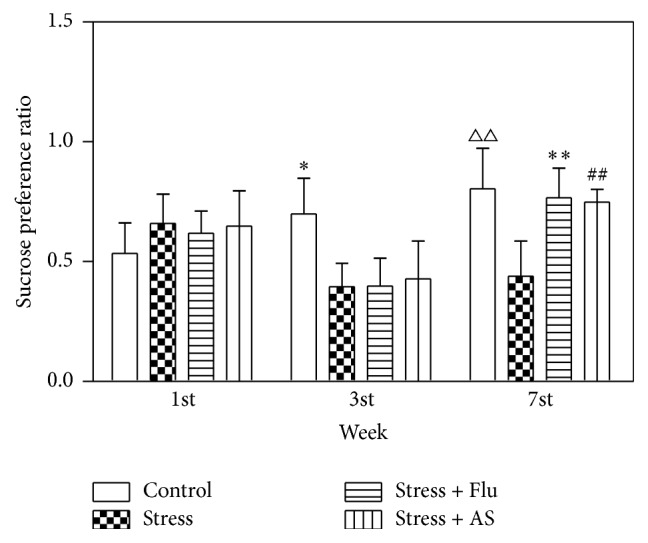
Effects of AS on CUMS induced depression on sucrose preference ratio. All results were represented as mean ± SD (*n* = 8 for each group). ^*∗*^ *p* < 0.05 compared with stress group. ^*∗∗*^ *p* < 0.01 compared with stress group. ^##^ *p* < 0.01 compared with stress group. ^△△^ *p* < 0.01 compared with stress group.

The results were shown in Figure 3. At the beginning of the experiments, there was no significant difference in the sucrose preference ratio across the groups. After two weeks of CUMS procedure, the sucrose preference ratio was clearly lower in the model group rats than in the control group rats (*p* < 0.05). After three weeks of treatment with fluoxetine treatment (10 mg/kg) or AS (1 g/kg), there was a significant difference between rats in AS and CUMS groups that demonstrated that the effect of AS was superior (*p* < 0.01). However, no statistical difference was found between the AS group and fluoxetine group (*p* > 0.05).

### 3.3. Effects of AS on Open Field Tests

**Figure 4:**
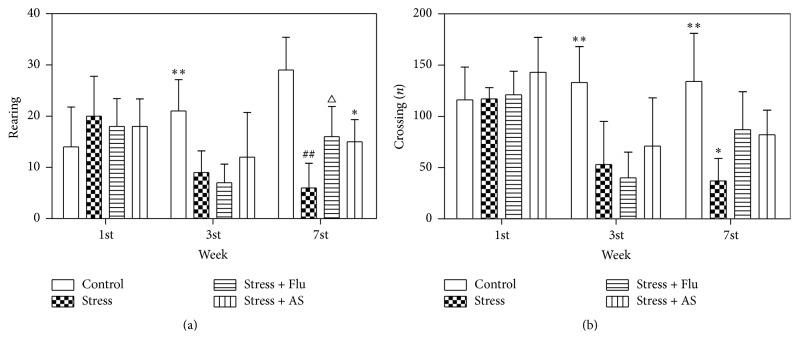
(a) Effects of AS on CUMS induced depression on rearing numbers. All results were represented as mean ± SD (*n* = 8). ^*∗∗*^ *p* < 0.01 compared with stress group. ^##^ *p* < 0.01 compared with control group. ^△^ *p* < 0.05 compared with stress group. ^*∗*^ *p* < 0.05 compared with stress group. (b) Effects of AS on CUMS induced depression on crossing numbers. All results were represented as mean ± SD (*n* = 8). ^*∗∗*^ *p* < 0.01 compared with stress group, fluoxetine group, and AS group. ^*∗*^ *p* < 0.05 compared with fluoxetine group and AS group.

The results were shown in Figure 4. We found that the experimental group of rats that were subjected to the CUMS procedure for two consecutive weeks showed fewer crossing (*p* < 0.01) and rearing numbers (*p* < 0.01) than the control group rats in open field tests (Figures 4(a) and 4(b)). More crossing (*p* < 0.05) and rearing numbers (*p* < 0.05) were exhibited by the rats that were treated with AS for three weeks (1 g/kg) than the control rats (Figures 4(a) and 4(b)). The positive control drug, fluoxetine (10 mg/kg), had a similar effect on the numbers of crossing and rearing behavior.

### 3.4. Effects of AS on Forced Swim Tests

**Figure 5:**
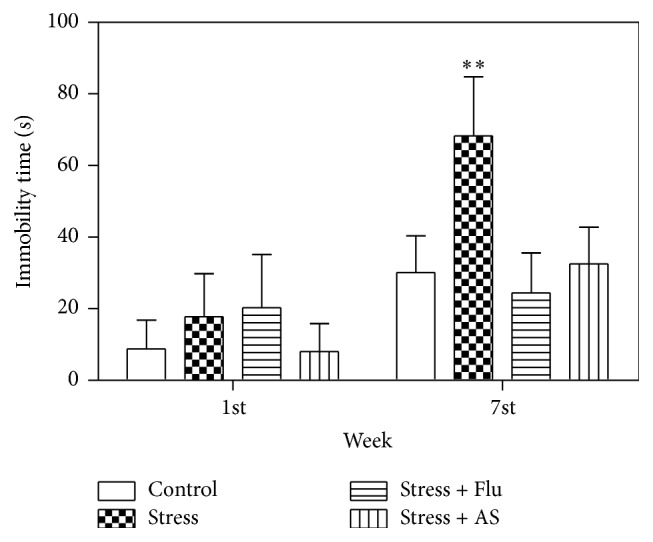
Effects of AS on CUMS induced depression on immobility time. All results were expressed as mean ± SD (*n* = 8). ^*∗∗*^ *p* < 0.01 compared with control group, fluoxetine group, and AS group.

The results of the forced swim tests are shown in Figure 5. There was no statistical difference in time spent immobile across all groups of rats at the beginning of the experiment (*p* > 0.05). However, immobility time was clearly longer in the rats treated with the CUMS procedure for 5 weeks than in the control group (*p* < 0.01). The rats treated with 3 weeks of AS (1 g/kg) or fluoxetine (10 mg/kg) spent less time immobile than the rats in the CUMS-only group (*p* < 0.01).

### 3.5. Effect of AS on BDNF Expression in the Hippocampus

**Figure 6:**
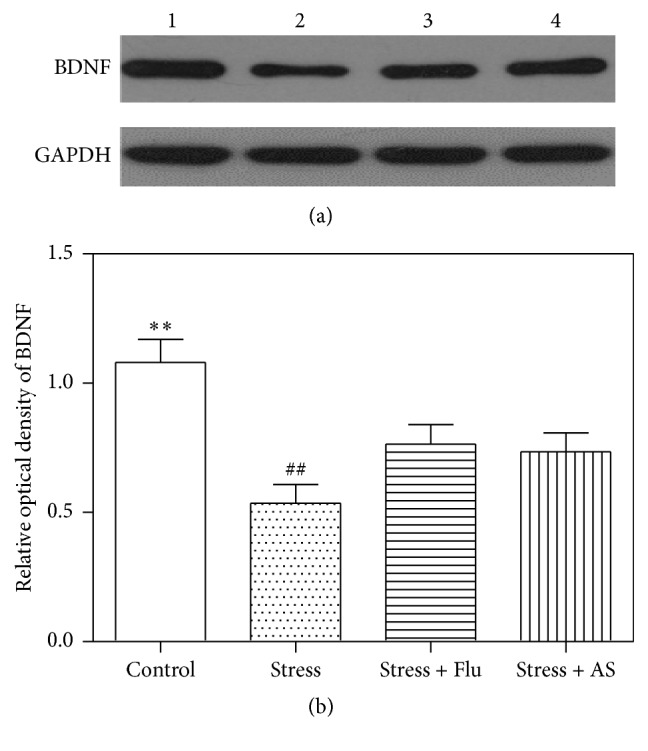
Effects of AS on CUMS induced depression on BDNF protein expression in hippocampus. (a) Western blot band. Band 1: control; Band 2: stress; Band 3: stress + fluoxetine; Band 4: stress+ AS. (b) Relative optical density of BDNF in the 4 groups. All results were expressed as mean ± SD (*n* = 6). ^*∗∗*^ *p* < 0.01 compared with fluoxetine group and AS group. ^##^ *p* < 0.01 compared with fluoxetine group and AS group.

As shown in Figure 6, hippocampal BDNF levels were measured at the end of experiment. The level of BDNF protein expression in the hippocampus was significantly lower in the rats that were exposed to 5 weeks of the CUMS procedure than in the control rats. We also found that treatment with AS and fluoxetine markedly reversed this effect on BDNF protein expression in the hippocampus (*p* < 0.01) but did not return it to normal levels. Additionally, there was a significant difference in BDNF expression between the AS, fluoxetine, and control groups (*p* < 0.01).

### 3.6. Effect of AS on CREB Phosphorylation in the Hippocampus

**Figure 7:**
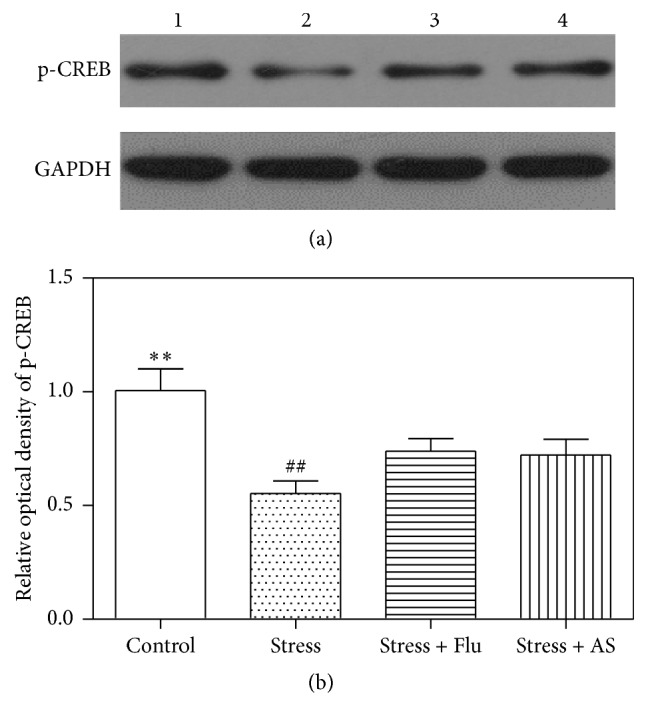
Effects of AS on CUMS induced depression on CREB phosphorylation protein expression in hippocampus. (a) Western blot band. Band 1: control; Band 2: stress; Band 3: stress + fluoxetine; Band 4: stress + AS. (b) Relative optical density of p-CREB in the 4 groups. Values were expressed as mean ± SD (*n* = 6). ^*∗∗*^ *p* < 0.01 compared with fluoxetine group and AS group. ^##^ *p* < 0.01 compared with fluoxetine group and AS group.

As shown in Figure 7, the CUMS procedure resulted in clearly lower levels of CREB phosphorylation in the hippocampus than were observed in the control rats (*p* < 0.01). After three weeks of treatment with AS (1 g/kg) and fluoxetine (10 mg/kg), hippocampal CREB phosphorylation was upregulated. There was significant difference between the AS (1 g/kg), fluoxetine (10 mg/kg), and model groups (*p* < 0.01).

### 3.7. Effect of AS on ERK 1/2 Phosphorylation in the Hippocampus

**Figure 8:**
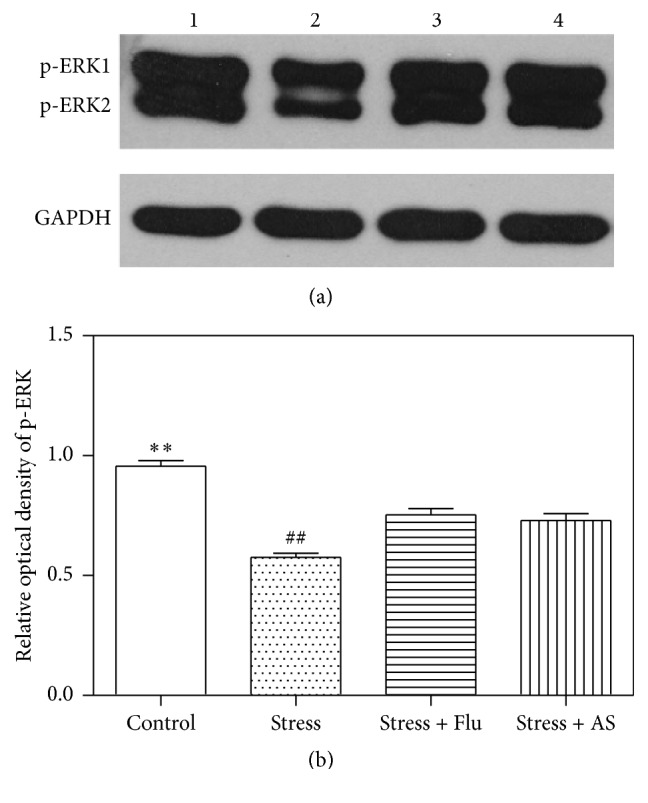
Effects of AS on CUMS induced depression on ERK 1/2 phosphorylation protein expression in hippocampus. (a) Western blot band. Band 1: control; Band 2: stress; Band 3: stress + fluoxetine; Band 4: stress + AS. (b) Relative optical density of p-ERK 1/2 in the 4 groups. All values were expressed as mean ± SD (*n* = 6). ^*∗∗*^ *p* < 0.01 compared with fluoxetine group and AS group. ^##^ *p* < 0.01 compared with fluoxetine group and AS group.

As shown in Figure 8, the hippocampal levels of phosphorylated ERK 1/2 were lower in the experimental rats than in the control rats (*p* < 0.01). Treatment with AS (1 g/kg) and fluoxetine (10 mg/kg) resulted in a higher level of hippocampal phosphorylated ERK 1/2 than was observed in the model rats (*p* < 0.01).

## 4. Discussion

In this study, we investigated the antidepressant effects of AS extracts on CUMS induced depression in rats. Treatment with AS extracts resulted in significantly higher rates of sucrose consumption, higher sucrose preference ratios in sucrose preference tests, more locomotor activity in open field tests, and less time spent immobile in forced swim tests. The antidepressant mechanism of AS extracts appears to involve a reversal of the CUMS-mediated decrease in the protein levels of hippocampal BDNF, p-CREB, and p-ERK 1/2.

The CUMS animal model has good validity when used to mimic people facing unpredictable pressure every day. It was widely used to induce depression in rodents and to screen antidepressants. This depression model was considered to be successful after it was applied for two consecutive weeks because we observed fewer crossing behaviors and an increase in sucrose consumption. Three weeks' treatment with fluoxetine and AS reversed these behavioral effects.

Rats subjected to CUMS consumed less sucrose and exhibited lower sucrose preference ratios, which was interpreted as anhedonia. Anhedonia is one of the core symptoms of depression and it has been previously recognized that human depressed individuals show reduced responses to rewards. The depressed rats that were given antidepressant or AS extracts consumed more sucrose than the CUMS rats; these results are in alignment with previous results [32]. In a novel environment, depressed rats displayed fewer locomotor activities in open filed tests, indicating a loss of interest in external stimuli. In the forced swim tests, the control rats tried their best to escape their surroundings, and their immobility times were significantly shorter than the times observed in the model rats. Moreover, the CUMS rats exhibited less struggling and more floating while in the container, indicating behavioral despair. Depressed patients do not deal positively with stressful events in daily life, and they also show behavioral despair. In this study, these behavioral symptoms were simulated by CUMS and reversed by treatment with fluoxetine and AS extracts. Moreover, the advantage of using the forced swim tests is that it allows researcher to screen and appraise the antidepressant effects of new drugs. The rats in the stress group gained less body weight than the rats in the control group, but there was no statistical difference between the AS, fluoxetine, and model groups. These results are in line with some studies, while others have shown that antidepressants can reverse body weight gain when results are compared to the stress-only group [33–35].

The mechanisms underlining depression remain poorly understood. In addition to the monoamine hypothesis, the neurotrophic hypothesis has received a growing amount of attention in recent years. Our results are consistent with previous studies in which BDNF expression and the phosphorylation of ERK and CREB were reduced in the hippocampus in rats that were exposed to CUMS, and each of these factors was upregulated by treatment with AS extracts [36]. BDNF is widely expressed throughout the central nervous system and peripheral system especially in hippocampus. BDNF plays a critical role in hippocampal neurogenesis, neuronal synaptic plasticity, and neuronal survival and differentiation. Some studies have confirmed that the BDNF signaling pathway is involved in CUMS induced depression [37, 38]. When BDNF binds to the ligand tropomyosin receptor kinase B, the resulting dimer can induce the phosphorylation of ERK and the downstream phosphorylation of CREB. Phosphorylated CREB enhances the transcription of some target genes, including BDNF, and promotes the protein expression of BDNF. Hippocampal atrophy is always observed in major depressive patients. Injuries to hippocampal neuronal synaptic plasticity and neuronal apoptosis eventually induce hippocampal shrinkage. The BDNF signaling pathway has been shown to be inhibited in rats subjected to CUMS, resulting in a reduction in hippocampal BDNF, p-ERK 1/2, and p-CREB protein levels. The chronic administration of antidepressants can result in a reversal of these molecular changes in the hippocampus, preventing hippocampal neuronal apoptosis and promoting synaptic plasticity. The symptoms of depression are thereby ameliorated.

## 5. Conclusions

In summary, we found that CUMS induced rats to display a depression state, and treatment with AS extracts exerted an antidepressant-like effect by activating the BDNF signaling pathway (BDNF-ERK 1/2-CREB) and upregulating the protein expression level of hippocampal BDNF and phosphorylated ERK 1/2 and CREB.

## Figures and Tables

**Table 1:** Experimental design.

Groups	1st week	2nd-3rd week	3rd weekend	4th–6th week	7th week
Control (*n* = 8)	Behavioral tests	SE (*n* = 8)	Sucrose preference & open field test	SE (*n* = 8)	Behavioral tests
Stress (*n* = 12)	Behavioral tests	CUMS (*n* = 12)	Sucrose preference & open field test	CUMS + water (*n* = 8)	Behavioral tests
Stress + Flu (*n* = 12)	Behavioral tests	CUMS (*n* = 12)	Sucrose preference & open field test	CUMS + Flu (*n* = 8)	Behavioral tests
Stress + AS (*n* = 12)	Behavioral tests	CUMS (*n* = 12)	Sucrose preference & open field test	CUMS + AS (*n* = 8)	Behavioral tests

SE: standard environment; CUMS: chronic unpredictable mild stress; behavioral tests: sucrose preference & open field & forced swim tests.

There are 44 rats in the start of experiment: control (*n* = 8), stress (*n* = 12), stress + Flu (*n* = 12), and stress + AS (*n* = 12).

The animal number of each group is eight in end stage.
